# Knowledge of General Dental Practitioners and Specialists about Emergency Management of Traumatic Dental Injuries in Qassim, Saudi Arabia

**DOI:** 10.1155/2020/6059346

**Published:** 2020-02-19

**Authors:** Sanaa N. Al-Haj Ali, Somaya A. Algarawi, Atheer M. Alrubaian, Asma I. Alasqah

**Affiliations:** ^1^Department of Orthodontics and Pediatric Dentistry, College of Dentistry, Qassim University, Qassim, Saudi Arabia; ^2^College of Dentistry, Qassim University, Qassim, Saudi Arabia

## Abstract

*Context*. Studies about knowledge of emergency management of traumatic dental injuries (TDIs) which affect children by general dental practitioners (GDPs) and specialists in Saudi Arabia are lacking.

**Aim:**

The aim of this cross-sectional study was to assess the knowledge level of GDPs and specialists about TDI emergency management and its relation with demographic variables in Qassim region, Saudi Arabia. *Materials and Methods*. A random sample of 239 GDPs and specialists was given a two-part questionnaire; the first part included demographic questions and the second part included questions related to knowledge of emergency management of luxation (intrusion and extrusion), complicated crown fracture, and avulsion injuries. Data was statistically analyzed using chi-square and ordinal logistic regression tests. The significance was set at *P* < 0.05.

**Results:**

The mean knowledge score was 5.57 for GDPs and 6.69 for specialists (out of 12). A significant difference was observed between both groups in the management of avulsion injury. Three factors significantly improved the dentists' knowledge: gender (female), practice type (specialist), and previous experience of encountered TDIs (*P* < 0.05.

**Conclusions:**

GDPs and specialists in Qassim region had moderate knowledge of emergency management of TDIs. Specialists were significantly more knowledgeable than GDPs in the management of avulsion injury when compared to the rest of the injuries.

## 1. Introduction

At present, traumatic dental injuries (TDIs) are recognized as a public dental health problem, especially in children and adolescents [1]. It is reported that 30% of children sustain injuries to the primary dentition and 22% to the permanent dentition [2]. It was also estimated that over 50% of children would sustain a TDI before leaving school [3].

The general consensus on TDI management is that patients should go directly to a hospital and be evaluated by a dentist [4]. In Saudi Arabia, dentists who are targeted to the general population are based in Ministry of Health facilities, universities, and the private sector [5]. However, emergency treatment is commonly provided by dentists who work in ministry of health centers/hospitals and private hospitals/clinics. Both general dental practitioners (GDPs) and specialists may provide emergency treatment of TDIs in these facilities. Therefore, their knowledge and skills in the management of TDIs in children should be adequate [6] as initial management if handled inappropriately may affect the prognosis of many TDIs [7].

Several international surveys demonstrated inadequate knowledge of dentists about emergency management of TDIs [6, 8–13]. Two studies investigated this topic in Saudi Arabia; however, the prime focus of these studies was to assess the knowledge level of dentists about emergency management of avulsion injury [14, 15]. As TDIs include other injuries and both GDPs and specialists in various dental disciplines can encounter such injuries in their practice, they must have sufficient knowledge on the emergency management of TDIs in general. The aim of this study was to assess the knowledge level of GDPs and specialists about TDI emergency management and its relation with demographic variables in Qassim region, Saudi Arabia.

## 2. Materials and Methods

The study population of this cross-sectional study consisted of a random sample of 239 dentists who served in dental centers/hospitals equipped with emergency services from the biggest three cities and six rural cities of Qassim region. A modified questionnaire [6, 11] was administrated to the dentists by the authors in the period from July to October 2017 and then collected. All participating dentists signed a consent form, and they were assured of strict confidentiality. This study was approved by the ethical committee of college of dentistry—Qassim University (reference number: EA/450/2017), and did not require financial support.

The questionnaire consisted of two parts. Part one comprised demographic questions about age, gender, nationality, type of practice (GDP/specialist), and if specialist which dental specialty, practice sector, years of experience, TDI first aid training, and encountered cases. Part two comprised 12 questions related to knowledge of emergency management of luxation injuries (intrusion and extrusion), complicated crown fracture, and avulsion injuries (Table 1).

Data were statistically analyzed using the SPSS computer software. Simple frequency distributions of dentists' answers were produced for part one questions, and for part two of the questionnaire, percentages of dentists' answers were also produced for part two questions and compared using a chi-square test. A knowledge score (0-12) was given to dentists according to their answers on part two of the questionnaire where every correct answer equaled one point. Ordinal logistic regression analysis was then used to identify the association of dentists' demographic data with the mean knowledge score. The level of significance was set at 0.05.

## 3. Results

The questionnaires were completed and returned by 239 (all) dentists, 172 of whom were GDPs and 67 were specialists. The mean knowledge score of GDPs was 5.57 and of specialists was 6.69. No significant difference was observed between the knowledge score of specialists regardless of their dental specialty. No significant differences were found between GDPs and specialists in the questions which concerned emergency management of luxation or complicated crown fracture (Tables 2 and 3) (*P* > 0.05); around 60% of specialists and 50% of GDPs chose the correct answer in case of intruded primary tooth, while slightly less and almost equal percentage of both groups (45%) knew that they should do the same (spontaneous repositioning) in case of intruded immature permanent tooth. More specialists than GDPs knew the proper splinting in case of a mature extruded tooth (47.8% vs. 38.4%). Also, more specialists knew the correct type of splint in that case (38.8% vs. 32.6%).

In complicated crown fracture questions, more than two-thirds of specialists (62.7%) and 50.6% of GDPs chose the correct answer (pulp capping) in case of pinpoint exposure of immature permanent tooth <3 hours of trauma, while partial pulpotomy was chosen by 29.9% of specialists and 26.2% of GDPs when the exposure was large >24 hours of trauma. The great majority of both groups (79.1% vs. 75.6%) knew the correct answer (pulpectomy) when the tooth was mature with large exposure >24 hours of trauma (Table 3).

Table 4 shows the distribution of dentists' answers about emergency management of tooth avulsion. The significant difference was found between both groups in the question which concerned the need for antibiotic following replantation (*P* = 0.048); more than two-thirds of specialists (65.7%) knew the correct answer compared to 50% of GDPs. More than two-thirds of specialists and GDPs (62.7% vs. 64.5%) chose the correct storage medium. On the other hand, 34.4% of specialists chose to soak the immature avulsed tooth <60 min of trauma in doxycycline for 5 min before replantation compared to 17.4% of GDPs. More specialists knew that avulsed primary teeth should not be replanted than GDPs (85.1% vs. 77.9%).

Table 5 shows the distribution of dentists according to demographic data and the results of ordinal logistic regression. Three factors significantly affected the knowledge score of the dentists; being a female dentist (*P* = 0.032), holding a specialty in dentistry and previous experience with TDIs (*P* < 0.001); female dentists were almost twice more likely to answer knowledge questions correctly and score higher than male dentists. Specialists were three times more likely to score higher than GDPs, while dentists who experienced TDIs during their practice were 2.6 times more likely to score higher than those without experience.

## 4. Discussion

In this study, the mean knowledge score of specialists (regardless of their specialty) about TDI emergency management was better than that of GDPs (6.69 vs. 5.57 out of 12). Nevertheless, the reported scores in this study are still within the moderate knowledge zone. These findings do not seem surprising in Saudi Arabia as the same findings about knowledge of emergency management of avulsion by dentists in Riyadh were reported by AlJazairy et al. [14] (score 5.94 out of 10) with an insignificant effect of specialty type on knowledge score. Al-Shamiri et al. [16] also reported insufficient knowledge concerning TDI management among dental students.

What actually improved the knowledge score of specialists from that of GDPs in this study is that they had sufficient and better knowledge of emergency management of avulsion injury than GDPs when compared to the rest of injuries as a significant difference between both groups was found in the management of avulsion. The only question which was answered correctly by less than half of the specialists was the one which concerned soaking the immature avulsed permanent tooth in doxycycline for 5 min before replantation. Although Cınar et al. [11] reported similar percentage of dentists (39.6%) choosing this management option, it seems that the importance of doxycycline in improving the prognosis and enhancing revascularization of avulsed immature teeth as reported by Trope [17] needs to be reinforced.

When knowledge of dentists about emergency management of luxation injuries was assessed, specialists were more aware than GDPs about the proper management of intruded primary teeth; however, their knowledge regarding proper management of immature permanent teeth did not seem satisfactory and different from that of GDPs as less than half of both groups were able to identify the correct answer. The same finding was reported by Cınar et al. [11]. Specialists were also more aware than GDPs, although to a lesser extent, in proper splinting and type of splint for extruded mature permanent teeth; however, it is worthy to mention that in these questions correct answers were reported by less than half of the specialists; consequently, their knowledge can be considered inadequate.

In this study, insufficient knowledge of emergency management of complicated crown fracture of immature permanent teeth with large exposure presenting very late was observed by both groups when compared to the rest of the questions. A correct answer in that question was chosen by 30% only of both groups. Apparently, dentists feel less confident in providing emergency treatment of such complex case in immature teeth when compared to the rest of the situations, which was also reported by Cauwels et al. [18]. According to the International Association of Dental Traumatology (IADT) guidelines, if the size of exposure is large in immature or mature permanent teeth, the pulp has been open to bacterial contamination or too much time has elapsed; then partial pulpotomy is indicated while pulpectomy is indicated if the pulp becomes necrotic in cases of a complicated crown fracture of immature teeth [19].

In this study, gender (female), type of practice (specialist), and experience with TDI cases significantly improved the knowledge score of the dentists. Similar findings were reported in previous studies [12, 14, 20].

It is important to address the limitation in this study as the use of random sample may not sufficiently represent the entire population of dentists working in Qassim.

## 5. Conclusion

Dentists' knowledge of emergency management of TDIs in Qassim region of Saudi Arabia was moderate. Female dentists, specialists, and those with previous experience in TDIs were significantly more knowledgeable. With the introduction of mandatory continuing medical education (CME) license renewal requirements by the Saudi Commission of Health Specialties for all dental care providers, continuing dental education courses in dental traumatology if arranged can provide dentists with the knowledge required for TDI emergency management. In addition, improving courses covering dental traumatology in dental postgraduate education programs seem necessary in Saudi Arabia. These should consequently lead to better clinical management of TDIs and fewer complications from such injuries in children.

## Figures and Tables

**Table 1:** Questions related to knowledge of emergency management of TDI.

Q	Situation	Answer options
1	If an intruded primary maxillary anterior tooth has been displaced toward the labial bone plate	(a) The tooth is left for spontaneous repositioning
		(b) The tooth is immediately extracted
		(c) Do not know

2	If an immature permanent maxillary tooth has been intruded, the tooth should be	(a) Left alone for spontaneous repositioning
		(b) Repositioned orthodontically
		(c) Repositioned surgically
		(d) Extracted immediately
		(e) Do not know

3	If a mature permanent maxillary tooth has been extruded, the tooth should be immediately repositioned and stabilised using a…	(a) Rigid splint for 4 weeks
		(b) Rigid splint for 2 weeks
		(c) Semirigid splint for 2 weeks in conjunction with RCT
		(d) Semirigid splint for 2 weeks in conjunction RCT if pulp necrosis has occurred

4	What type of splint should be used for extruded permanent incisors?	(a) Semirigid with a nylon wire
		(b) Stainless steel wire
		(c) Composite resin
		(d) Other

5	If a patient with an immature permanent maxillary tooth injury with pinpoint pulp exposure came to the clinic within 3 hours after the trauma, the treatment procedure would be…	(a) Do not treat but follow up (b) Pulp capping (c) Partial pulpotomy (d) Cervical pulpotomy (e) Pulpectomy (f) Do not know
6	If a patient with an immature permanent maxillary tooth injury with large pulp exposure came to the clinic more than 24 hours after the trauma, the treatment procedure would be…	
7	If a patient with a mature permanent maxillary tooth injury with large pulp exposure came to the clinic more than 24 hours after trauma, the treatment procedure would be…	

8	Which of the following storage media are suitable for the storage of an avulsed tooth?	(a) Ice
		(b) Tap water
		(c) Paper tissue
		(d) Fresh milk
		(e) Patient's mouth

9	If the patient comes to the clinic within 60 min after trauma, before replantation, the immature avulsed tooth should be…	(a) Rinsed with tap water
		(b) Cleaned with any type of solution
		(c) Left unwashed
		(d) Kept in doxycycline for 5 min
		(e) Scrubbed gently
		(f) Kept in fluoride solution for 20 min
		(g) Do not know

10	If the patient came to the clinic more than 60 min after trauma, for what period do you indicate the use of a splint for a mature avulsed tooth?	(a) No splint
		(b) 2 weeks
		(c) 4 weeks
		(d) 2 months
		(e) 24 hours
		(f) Do not know

11	After replantation, do you prescribe antibiotic therapy?	(a) Yes, tetracycline (>12 years old)
		(b) Yes, penicillin
		(c) No

12	Should avulsed primary teeth be replanted?	(a) Yes
		(b) No

RCT: root canal treatment.

**Table 2:** Frequency distribution (%) of dentists' answers about emergency management of luxated teeth.

Situation	Answer	*N* (%)	
		Specialists	GDPs
(1) Intruded primary upper anterior toward the bone plate	(A) Spontaneous repositioning^∗^	40 (59.7)	83 (48.3)
	(B) Extraction immediately	24 (35.8)	78 (45.3)
	(C) Do not know	3 (4.5)	11 (6.4)

(2) Immature permanent intruded upper anterior	(A) Spontaneous repositioning^∗^	31 (46.3)	78 (45.3)
	(B) Orthodontic repositioning	19 (28.4)	49 (28.5)
	(C) Surgical repositioning	15 (22.4)	28 (16.3)
	(D) Extraction immediately	1 (1.5)	10 (5.8)
	(E) Do not know	1 (1.5)	7 (4.1)

(3) Mature permanent extruded upper tooth splinting	(A) Rigid splint for 4 weeks	6 (9)	21 (12.2)
	(B) Rigid splint for 2 weeks	5 (7.5)	31 (18)
	(C) Semirigid splint for 2 weeks with RCT	24 (35.8)	54 (31.4)
	(D) Semirigid splint for 2 weeks with RCT in case of pulp necrosis^∗^	32 (47.8)	66 (38.4)

(4) Type of splint for extruded permanent incisor	(A) Semirigid with nylon wire^∗^	26 (38.8)	56 (32.6)
	(B) Stainless steel wire	20 (29.9)	58 (33.7)
	(C) Composite resin	19 (28.4)	44 (25.6)
	(D) Other	2 (3)	14 (8.2)

^∗^Correct answer.

**Table 3:** Frequency distribution (%) of dentists' answers about emergency management of crown fracture.

Situation	(A) Immature permanent upper tooth, pinpoint exposure within 3 hours of trauma		(B) Immature permanent upper tooth, large exposure more than 24 hours after trauma		(C) Mature permanent upper tooth, large exposure more than 24 hours after trauma	
Answer	Specialists	GDPs	Specialists	GDPs	Specialists	GDPs
Only follow-up	6 (9)	6 (3.5)	0 (0)	2 (1.2)	2 (3)	3 (1.7)
Pulp capping^∗^	42 (62.7)	87 (50.6)	3 (4.5)	11 (6.4)	3 (4.5)	4 (2.3)
Partial pulpotomy^∗^	12 (17.9)	41 (23.8)	20 (29.9)	45 (26.2)	2 (3)	12 (7)
Cervical pulpotomy	3 (4.5)	14 (8.1)	15 (22.4)	39 (22.7)	6 (9)	16 (9.3)
Pulpectomy^∗^	2 (3)	21 (12.2)	28 (41.8)	69 (40.1)	53 (79.1)	130 (75.6)
Do not know	2 (3)	3 (1.7)	1 (1.5)	6 (3.5)	1 (1.5)	7 (4.1)

^∗^Correct answer in situations A, B, and C.

**Table 4:** Frequency distribution (%) of dentists' answers about emergency management of tooth avulsion.

Situation	Answer	*N* (%)	
		Specialists	GDPs
(1) Suitable storage medium for avulsed tooth	(A) Ice	2 (3)	2 (1.2)
	(B) Tap water	0 (0)	5 (2.9)
	(C) Paper tissue	2 (3)	6 (3.5)
	(D) Fresh milk^∗^	42 (62.7)	111 (64.5)
	(E) Patient's mouth	21 (31.3)	48 (27.9)

(2) Before replanting avulsed immature tooth within 60 min of trauma	(A) Rinse with tap water	17 (25.4)	42 (24.4)
	(B) Clean with any solution	9 (13.4)	22 (12.8)
	(C) Leave unwashed	4 (6)	16 (9.3)
	(D) Keep in doxycycline for 5 minutes^∗^	23 (34.4)	30 (17.4)
	(E) Scrub gently	7 (10.4)	28 (16.3)
	(F) Keep in fluoride for 20 minutes	3 (4.5)	19 (11)
	(G) Do not know	4 (6)	15 (8.7)

(3) Splinting period for mature avulsed tooth more than 60 min after trauma	(A) No splint	2 (3)	5 (2.9)
	(B) 2 weeks	24 (35.8)	78 (45.3)
	(C) 4 weeks^∗^	37 (55.2)	61 (35.5)
	(D) 2 months	2 (3)	14 (8.1)
	(E) 24 hours	1 (1.5)	5 (2.9)
	(F) Do not know	1 (1.5)	9 (5.2)

(4) Need for antibiotic following replantation	(A) Yes, tetracycline (>12 years)	11 (16.4)	42 (24.4)
	(B) Yes, penicillin^∗^	44 (65.7)	86 (50)
	(C) No	12 (17.9)	44 (25.6)

(5) Avulsed primary tooth should be replanted	(A) Yes	10 (14.9)	38 (22.1)
	(B) No^∗^	57 (85.1)	134 (77.9)

^∗^Correct answer.

**Table 5:** Frequency of dentists according to demographic data and association with knowledge score.

Variable	*N*	Mean knowledge score	EXP (B)	95% confidence interval		Sig.
				Lower	Upper	
Age (in years):						
20-30	84	6.04	2.899	0.689	12.193	0.147
31-40	100	5.63	1.765	0.471	6.610	0.399
41-50	44	6.32	3.399	0.934	12.370	0.063
>50	11	5.27	1	Referent		—

Gender:						
Male	132	5.78	0.579	0.352	0.955	0.032^∗^
Female	107	6.01	1	Referent		—

Nationality:						
Saudi	54	5.56	0.615	0.299	6.039	0.187
Non-Saudi	185	5.98	1	Referent		—

Type of practice:						
Specialist	67	6.69	3.156	1.759	5.660	<0.001^∗^
GDP	172	5.57	1	Referent		—

Practice sector:						
Public	72	5.67	0.746	0.423	1.316	0.312
Private	167	5.98	1	Referent		—

Years of experience:						
<5	56	6.29	1.944	0.555	6.809	0.299
6-10	80	5.67	1.044	0.350	3.108	0.939
11-20	82	5.84	1.074	0.387	2.983	0.891
>21	21	5.76	1	Referent		—

Training about TDI emergency treatment:						
Yes	152	5.95	0.937	0.569	1.542	0.797
No	87	5.77	1	Referent		—

Encountered cases of TDIs:						
Yes	175	6.12	2.668	1.544	4.611	<0.001^∗^
No	64	5.23	1	Referent		—

^∗^Statistically significant. GDPs: general dental practitioners; TDIs: traumatic dental injuries.

## Data Availability

The data used to support the findings of this study are available from the corresponding author upon request.
